# Approach and Treatment of Giant Luminal Unicystic Ameloblastoma

**DOI:** 10.1155/2018/6809758

**Published:** 2018-05-30

**Authors:** George Borja de Freitas, Evelyne Pedroza de Andrade, Riedel Frota Sá Nogueira Neves, Stefanny Torres dos Santos, Daniella Cristina da Costa Araújo, Victor Ângelo Montalli

**Affiliations:** ^1^Department of Oral Pathology, São Leopoldo Mandic Institute and Research Center, Campinas, SP, Brazil; ^2^Department Maxillofacial Surgery, Hospital Getúlio Vargas, Recife, PE, Brazil

## Abstract

Unicystic ameloblastoma is an odontogenic tumor that affects mainly young patients and usually involves the posterior region of the mandible. In this article, we report on the case a 12-year-old girl presenting with an 8-month history of facial swelling in her lower right quadrant. Radiographic examination revealed a unilocular radiolucent lesion extending from the body of the mandible through to the angle and ascending ramus. An incisional biopsy was performed, and a diagnosis of luminal unicystic ameloblastoma was made based on clinicopathological features. The lesion was treated in two stages, namely, an initial conservative approach via decompression and subsequent excision. The patient has been followed up for 6 months without clinical and radiographic evidence of recurrence. In conclusion, conservative timely intervention combined with a conservative surgical approach has proven efficacious in the treatment of ameloblastoma in this young patient.

## 1. Introduction

Unicystic ameloblastoma is a cystic lesion that shows clinical and radiographic features that resemble those of odontogenic cysts, though histological features are crucial to ascertain the presence of ameloblastic epithelium and its variants [1].

This lesion mainly affects the posterior regions of the mandible in young patients. Its radiographic features include a single unilocular and radiolucent lesion generally associated with the crown of an unerupted third molar [1].

Such lesions tend to grow slowly, though locally invasive, which may cause bone deformities and painless swelling [2, 3]. They may be classified into three histological variants: luminal, intraluminal, and mural. Treatment planning and prognosis are based on the histological subtype [1, 3].

Several treatment approaches have been reported, such as enucleation, marsupialization, and resection of the affected area; however, conservative measures tend to be preferred in young patients [4].

## 2. Case Report

**Figure 1:**
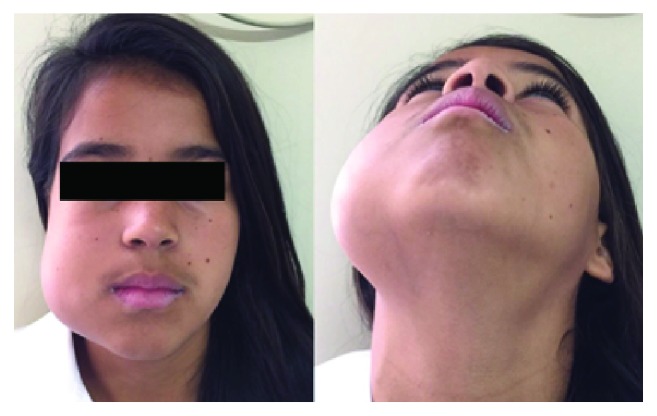
A 12-year-old Brazilian female with a chief complaint of a painless growing facial swelling for 8 months.

**Figure 2:**
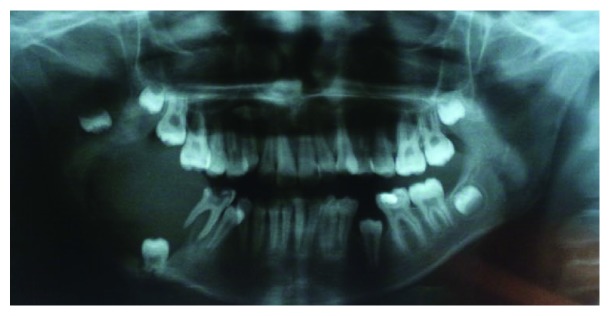
Panoramic radiographic examination revealed a unilocular radiolucent lesion extending from the body of the mandible through to the angle and right ascending ramus.

**Figure 3:**
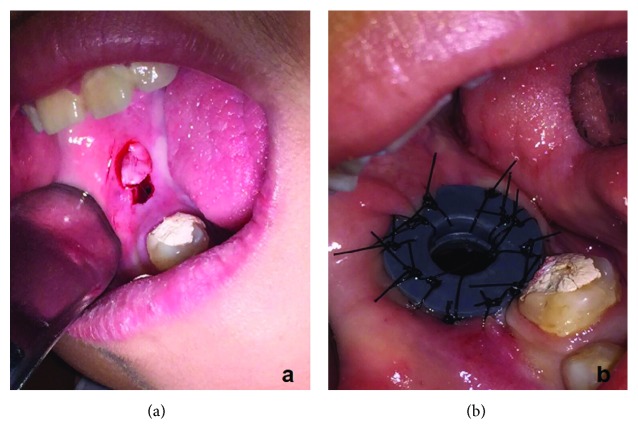
Incisional biopsy (a). Flexible tube for decompression (b).

**Figure 4:**
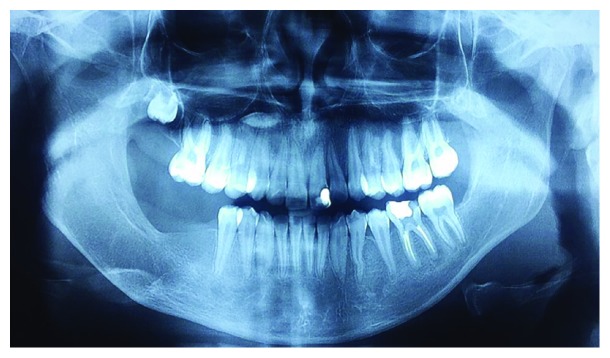
The patient has been followed up for 6 months, with no clinical or radiographic evidence of recurrence.

A 12-year-old Brazilian female, with no systemic comorbidities, attended the oral and maxillofacial surgery service with a chief complaint of a painless growing facial swelling for 8 months (Figure 1). On extraoral examination, a unilateral expansive lesion was detected on the lower right aspect of her face. On intraoral examination, the swelling could be seen affecting the body and the angle of the mandible on the right side associated with her lower right third molar. Panoramic radiographic examination revealed a unilocular radiolucent lesion extending from the body of the mandible through to the angle and right ascending ramus, causing displacement of the second molar towards the base of the mandible and the third molar towards the ascending ramus (Figure 2). A CT scan revealed that the lesion caused expansion of the buccal and lingual aspects of the cortical bone with areas of fenestration. Needle aspiration was performed to evaluate the contents of the swelling, mainly to exclude the possibility of a vascular lesion. An incisional biopsy was then performed, and the specimen was sent for histological evaluation (Figure 3). The access window left from the biopsy was used to accommodate a flexible tube for decompression and subsequent volume reduction of the lesion in an attempt to minimize the need for mutilating surgery in such a young patient. In view of the clinical and radiographic characteristics, two differential diagnoses were raised, namely, unicystic ameloblastoma or dentigerous cyst (Figure 3). The histological diagnosis confirmed the suspicion of a unicystic ameloblastoma. A decision was made to continue with the assisted decompression approach using daily irrigations of sterile saline solution intercalated with 0.12% chlorhexidine digluconate to remove debris and decontaminate the site, which was followed up both clinically and radiographically. After 5 months of decompression, a significant reduction of the lesion was observed radiographically, with evidence of bone neoformation in the periphery of the lesion. In view of the favorable progression, complete enucleation of the lesion combined with peripheral osteotomy and cryotherapy was performed under general anesthesia to reduce the risk of recurrence. The excised specimen was sent for histopathological evaluation, which reiterated the previous diagnosis of unicystic ameloblastoma. The patient has been followed up for 6 months, with no clinical or radiographic evidence of recurrence (Figure 4). A supernumerary tooth in the right maxilla was also observed in the panoramic radiograph. This tooth was not removed since cone beam computed tomography was not available for a better surgical planning due to financial reasons. However, the patient remains in close follow-up.

**Figure 5:**
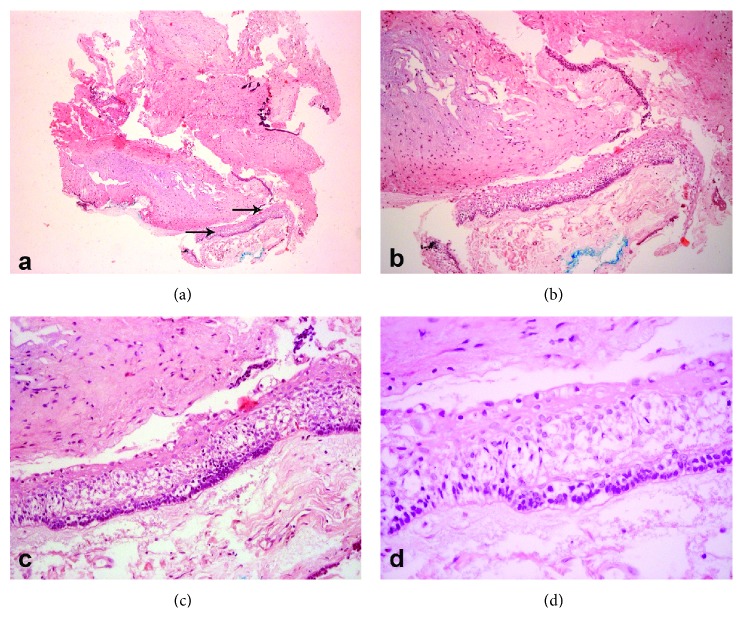
Photomicrograph showing ameloblastic epithelium. Hematoxylin and eosin. Original magnification 40x (a). Original magnification 100x (b). Original magnification 200x (c). Original Magnification 400x (d).

The histological sections showed the presence of a fibrous capsule lined by nonkeratinized stratified pavement epithelium exhibiting spongiosis, reverse polarization of the basal layer, and areas that resembled the stellate reticulum. The fibrous capsule consisted of dense connective tissue, presenting moderate to severe lymphoplasmacytic inflammatory infiltrate and hemorrhagic areas (Figure 5).

## 3. Discussion

Unicystic ameloblastoma usually presents as slow growing, persistent, and locally invasive lesions, which may lead to bone deformation [1]. These expansive lesions exhibit well-defined radiolucent areas, which are surrounded by sclerotic borders, mainly in the posterior region of the mandible [5]. In this report, a unilocular radiolucent lesion was clearly outlined extending from the body of the mandible, through to the angle and ascending ramus, causing displacement of the teeth 47 and 48, which corroborates the descriptions for this type of lesions in the literature.

Most such cases are diagnosed in the second decade of life [1, 2, 5]. Painful lesions are associated with older patients and do not seem to correlate directly with tumor growth [5]. Thus, as in this report, a young patient (12 years old), asymptomatic, exhibiting root resorption corroborated previous published reports describing a mean age of 13 years at diagnosis, predominantly affecting the mandible [1].

Unicystic ameloblastomas may present three histological variants, where the luminal subtype of the tumor is confined to the luminal surface of the cyst and the cystic wall is totally or partially lined by ameloblastic epithelium; the intraluminal variant presents ameloblastoma nodules protruding into the lumen of the cyst and finally, in the third variant, known as mural, the cystic wall is infiltrated by ameloblastoma [5, 6]. The histological characteristics described on the excised specimen of the present case are compatible with the luminal subtype.

Despite there being a consensus that ameloblastomas should be treated radically to prevent recurrence, one is often faced with a dilemma whenever the treatment plan involves children, since the mandibular lesions at this age are usually benign, and, therefore, conservative surgery should be the treatment of choice, especially in a context of bone growth and unerupted teeth [1, 7–9].

Still regarding management, it is worth mentioning enucleation, marsupialization followed by enucleation, enucleation and chemical cauterization (Carnoy's solution), cryotherapy, decompression followed by enucleation, and peripheral osteotomy. Indeed, rare are the cases for which block resection has been the treatment of choice [1]. Such findings are pertinent with the approach selected for the case presented herein, where decompression was performed using a flexible device installed on the occasion of the incisional biopsy, followed by enucleation and peripheral osteotomy, in order to minimize the risk of recurrence. The patient has been followed up for six months without evidence of relapse.

## 4. Conclusion

Conservative timely intervention and conservative surgery combined with local adjuvants such as liquid nitrogen cryotherapy, peripheral osteotomy, and exodontia should be the first treatment option for ameloblastoma in young patients. Such an approach has often been associated with positive outcomes, including from the esthetic, psychological, and functional viewpoints, when compared to major resections.
